# Synovial Metastasis From Urothelial Carcinoma of the Renal Pelvis Causing Recurrent Hemarthrosis: A Rare Presentation

**DOI:** 10.7759/cureus.36983

**Published:** 2023-03-31

**Authors:** Rachel Tian Shian Wang, Qingyuan Zhuang, Xiu Fen Chen

**Affiliations:** 1 Advanced Internal Medicine, Singapore General Hospital, Singapore, SGP; 2 Supportive and Palliative Care, National Cancer Centre Singapore, Singapore, SGP; 3 Anatomical Pathology, Singapore General Hospital, Singapore, SGP

**Keywords:** palliative radiation therapy, malignant synovitis, arthrocentesis, urothelial cancer, synovial metastasis, recurrent hemarthrosis

## Abstract

Synovial metastases are rare for any malignancy. This case report discusses a case of synovial metastasis from urothelial carcinoma of the renal pelvis presenting with recurrent hemarthrosis. The diagnosis of malignant synovitis can be obtained by synovial fluid aspiration, which is a quick and minimally invasive method, especially when imaging is unyielding or unspecific. Unfortunately, the diagnosis is associated with a poor prognosis of about five months, and treatment is often palliative. While no clinical guidelines exist, a multimodal and multidisciplinary management approach can help address the physical and psychosocial losses suffered.

## Introduction

Upper tract urothelial carcinoma, located in the pyelocaliceal cavities or ureter, is relatively rare, accounting for about 5% of urothelial carcinomas [1]. The most common sites of metastases are lymph nodes, lung, liver, bone and peritoneum [2]. The presence of distant metastasis often confers a poor prognosis [3]. To our knowledge, only a single case of synovial metastasis in transitional cell carcinoma of the bladder has been described [4]. We discuss a case of synovial metastasis from urothelial carcinoma of the renal pelvis, presenting with recurrent hemarthrosis.

## Case presentation

A 66-year-old man was diagnosed with high-grade invasive papillary urothelial carcinoma of the right renal pelvis in January 2018. He underwent a right nephroureterectomy and subsequent left retrograde intrarenal surgery, laser ablation, and ureteric stenting when the disease progressed. It metastasized in 2019, and he was placed on systemic therapy with palliative intent. Despite multiple lines of systemic therapy, including five cycles of nivolumab and ipilimumab under trial, 12 cycles of gemcitabine and carboplatin, four cycles of paclitaxel, and two cycles of docetaxel, his disease progressed. He also underwent radiotherapy to the left hip and femur, left pelvic wall, and 7th cervical to 2nd thoracic vertebrae for pain relief. The following doses were administered: one fraction of 8 Gy to the left femur, six fractions of 2.4 Gy to the left hip, and six fractions of 2.4 Gy to the 7th cervical to 2nd thoracic vertebrae. This was further complicated by a pathological fracture of the proximal femur, status post intramedullary nailing in 2019. In addition, he was started on rivaroxaban in November 2021 for an incidental finding of left internal jugular vein thrombosis.

**Figure 1:**
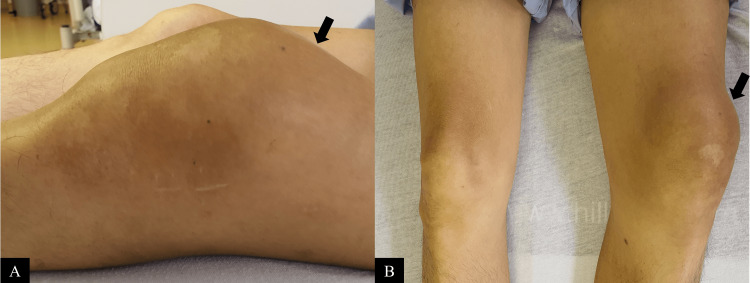
Photographs of (A) a lateral view of the left knee showing a large effusion and (B) in comparison to the right knee.

In December 2021, he presented to the emergency department with a two-week history of acute severe left knee pain. There was no preceding fall or trauma. This was associated with an acute deterioration of Eastern Cooperative Oncology Group (ECOG) status from 0 to 4. On examination, there was a large left knee joint effusion that was diffusely tender (Figures 1A, 1B). There were no overlying skin changes or erythema. The rest of the examination was unremarkable.

Investigations 

**Figure 2:**
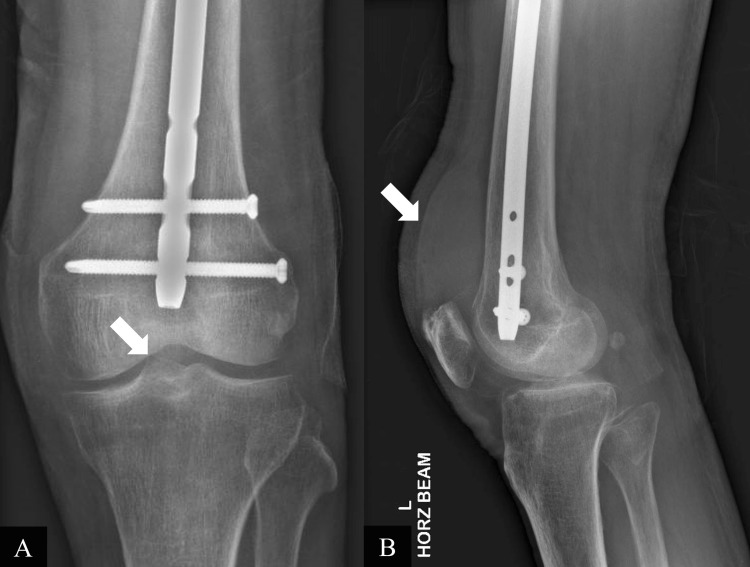
Anteroposterior (A) and lateral (B) radiographs of the left knee showing a hyperdense suprapatellar effusion with background patellofemoral and medial tibiofemoral compartment osteoarthritis.

He underwent a knee joint aspiration on the second day of admission, which yielded 150mL of heavily blood-stained fluid. Fluid gram stain and culture, mycobacterial smear and culture, and fungal culture were negative, ruling out septic arthritis. No crystals were seen on polarized microscopy. The synovial fluid was also sent for cytology. Plain radiographs of the left knee showed a hyperdense suprapatellar effusion with background patellofemoral and medial tibiofemoral compartment osteoarthritis (Figures 2A, 2B).

**Figure 3:**
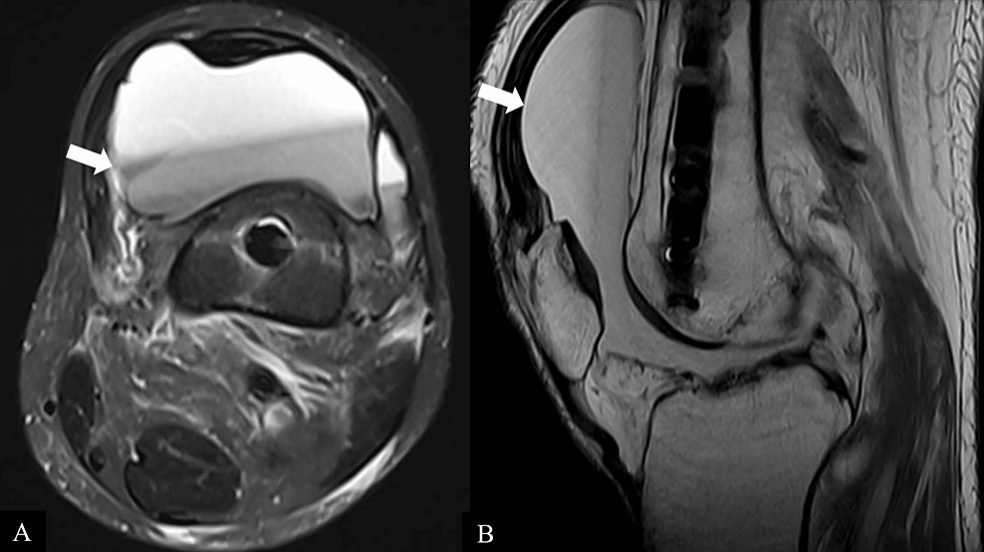
Axial (A) and sagittal (B) views of the left knee on magnetic resonance imaging (MRI) showing a large joint effusion with a fluid-fluid level, demonstrating largely T2-weighted hyperintense signal and intermediate T1-weighted signal suspicious for hemarthrosis.

Magnetic resonance imaging (MRI) of the left knee revealed a large joint effusion suspicious for hemarthrosis but no periprosthetic fracture, mass, synovial irregularity, or chondral defect, ruling out any traumatic causes or tumors. The joint and ligaments were intact (Figures 3A, 3B).

**Figure 4:**
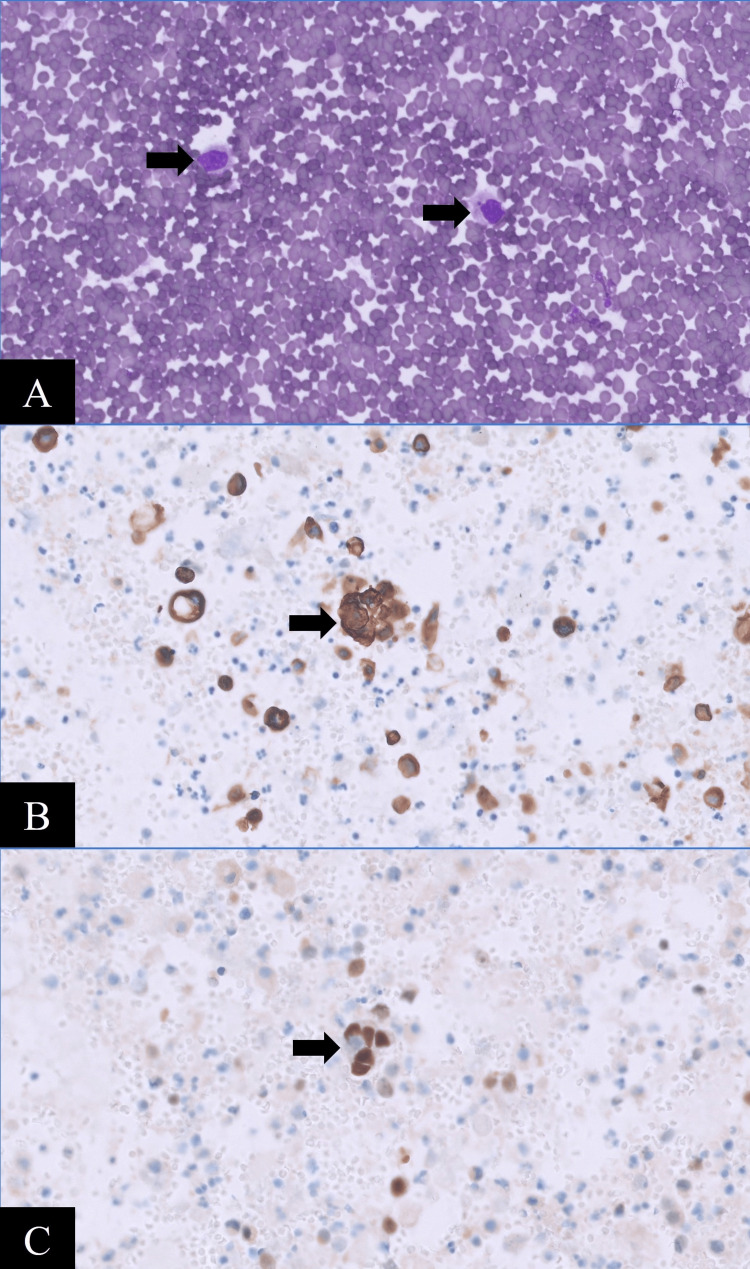
Photomicrographs of (A) Diff-Quik stain revealing singly scattered atypical cells with high nuclear-to-cytoplasmic ratio in a hemorrhagic background, and immunocytochemistry positive for (B) epithelial marker MNF116 and (C) transcription factor GATA-3, suggestive of urothelial origin.

A few days later, the painful left knee swelling recurred. Repeat therapeutic knee arthrocentesis obtained 175mL of bloody fluid. Again, microbiological studies were negative. A computed tomography (CT) angiography of the knee did not show vascular disorders such as bleeding aneurysms or any active bleeding suitable for angioembolization. Eventually, synovial fluid cytology revealed atypical cells positive for metastatic carcinoma. Cell block showed similar scattered atypical cells that expressed MNF116 and GATA3, suggesting metastatic synovial involvement of urothelial origin (Figures 4A-4C).

Treatment

A multidisciplinary approach was utilized to control symptoms and optimize function. The orthopedics team deemed that there was no role of left knee arthroscopic washout or total knee replacement in view of patient’s poor prognosis and operative risk. Repeated therapeutic knee aspirations were performed, on the fourth and fifth week of admission. Bleeding diathesis was considered, especially since the patient was on a direct oral anticoagulant. A hematology consult suggested that the initial re-accumulation of hemarthrosis may be due to the drug effect of rivaroxaban still being present. 500mL of fresh frozen plasma was transfused. However, this had no impact on the quick accumulation of hemarthrosis.

The patient had two fractions of 8 Gy external beam radiotherapy to the left knee for pain control and hemostasis. The dose was hypofractionated to reduce logistical burden in view of his poor performance status and prognosis. Together with the palliative team optimizing analgesia with opioids and adjuncts, the patient’s symptoms improved, although the knee effusion remained present. The medical social worker reviewed regularly for psycho-emotional support, and the physiotherapist and occupational therapist continued to assist in exercises and transfers. He was eventually competent in wheelchair mobilization with supervision.

For his underlying metastatic cancer, he was initiated on one cycle of pemetrexed, which was unfortunately complicated by neutropenic sepsis. Restaging scans showed disease progression and best supportive care was established. He was discharged home with hospice day care.

Outcome

Eventually, the patient passed away in April 2022 due to progression of his metastatic urothelial carcinoma, four months after presenting with malignant hemarthrosis. 

## Discussion

Synovial metastases are uncommon, with approximately only 50 case reports in the literature, and those originating from urothelial cancer are even more rarely seen. The majority of the cases reported in the literature originate from primary lung [5-8] and colorectal malignancies [6,9-11]. The clinical presentation is often that of chronic inflammatory arthritis. While the pathophysiology of synovial metastases remains unproven, it has been suggested to be either through direct extension from adjacent bone metastasis or through hematogenous spread into the subintimal synovial blood vessels [7,9,10,12], although the latter is less common [5,11,12]. Given the lack of adjacent osseous metastases in our case, a hematogenous spread may be postulated.

Early diagnosis is critical as it affects management and prognosis. Metastatic disease should be considered if a patient with underlying cancer presents with recurrent painful joint effusion, especially if commoner causes like crystal arthropathies, septic arthritis, and rheumatological disorders have been ruled out. In this case, the patient presented with recurrent hemarthrosis of the left knee joint, which was initially thought to be secondary to anticoagulant therapy, septic arthritis, or a bleeding disorder. However, the synovial fluid cytology results confirmed the diagnosis of synovial metastasis from urothelial carcinoma of the renal pelvis. We postulate that joint fluid analysis is crucial in clinching the diagnosis of malignant synovitis [4,7,9,10,13], especially if detailed imaging such as MRI or CT is unyielding. Most of the cases report the synovial fluid as hemorrhagic with few inflammatory cells, which tend to recur quickly after aspiration [6,8,9,11]. A synovial biopsy may be considered if the synovial fluid cytology is unyielding, and the index of suspicion remains high [14]. Further imaging with a CT, MRI, or bone scan has been described in previous cases to delineate any bony or soft tissue pathology [4-8,10,12,13].

The prognosis for patients with synovial metastasis is generally poor, with an average survival of approximately five months [4,5,8,12]. Treatment is often palliative and aims to improve the patient's quality of life by controlling pain and other symptoms. In this case, repeated arthrocentesis provided only temporary relief, while a combination of radiotherapy, analgesia, and physiotherapy helped the patient at least achieve wheelchair mobility and pain reduction.

There are no clinical guidelines for the management of synovial metastasis, and a multidisciplinary approach is required to address the physical and psychosocial losses suffered by patients with this condition. Overall, the management plan should be tailored to the individual patient's needs, considering factors such as the site and extent of metastasis, overall health, and treatment goals. A multimodal approach to optimizing pain control using analgesics and local radiotherapy may be most efficacious [4,10-12]. Palliative systemic chemotherapy and radiotherapy to treat the underlying cancer are also described in reported cases. Further support from our allied health staff including the medical social worker, physiotherapist, and occupational therapist is indispensable.

## Conclusions

Synovial metastases should be considered if a patient with underlying malignancy presents with recurrent synovitis. Synovial fluid aspiration is a quick and minimally invasive method of obtaining a cytopathological diagnosis of malignant synovitis and may help clinch the diagnosis, especially if imaging is unyielding or unspecific.

Unfortunately, the diagnosis confers a poor prognosis of approximately five months. Although no clinical guidelines currently exist, a multi-disciplinary and multimodal approach using analgesia, radiotherapy, and physiotherapy can ameliorate the highly symptomatic and debilitating nature of the disease.
